# Causality of genetically determined blood metabolites on irritable bowel syndrome: A Mendelian randomization study

**DOI:** 10.1371/journal.pone.0298963

**Published:** 2024-04-03

**Authors:** Xinyi Dai, Min Liang, Yanna Dai, Shaohua Ding, Xiaohe Sun, Luzhou Xu

**Affiliations:** 1 Nanjing University of Chinese Medicine, Nanjing, China; 2 Affiliated Hospital of Nanjing University of Chinese Medicine, Jiangsu Province Hospital of Chinese Medicine, Nanjing, China; 3 Department of Traditional Chinese Medicine, Wuxi Xinwu District Rehabilitation Hospital, Wuxi, China; Shenzhen Baoan Women’s and Children’s Hospital, CHINA

## Abstract

**Background:**

Irritable bowel syndrome (IBS) is one of the most common functional bowel disorders and dysmetabolism plays an important role in the pathogenesis of disease. Nevertheless, there remains a lack of information regarding the causal relationship between circulating metabolites and IBS. A two-sample Mendelian randomization (MR) analysis was conducted in order to evaluate the causal relationship between genetically proxied 486 blood metabolites and IBS.

**Methods:**

A two-sample MR analysis was implemented to assess the causality of blood metabolites on IBS. The study utilized a genome-wide association study (GWAS) to examine 486 metabolites as the exposure variable while employing a GWAS study with 486,601 individuals of European descent as the outcome variable. The inverse-variance weighted (IVW) method was used to estimate the causal relationship of metabolites on IBS, while several methods were performed to eliminate the pleiotropy and heterogeneity. Another GWAS data was used for replication and meta-analysis. In addition, reverse MR and linkage disequilibrium score regression (LDSC) were employed for additional assessment. Multivariable MR analysis was conducted in order to evaluate the direct impact of metabolites on IBS.

**Results:**

Three known and two unknown metabolites were identified as being associated with the development of IBS. Higher levels of butyryl carnitine (OR(95%CI):1.10(1.02–1.18),p = 0.009) and tetradecanedioate (OR(95%CI):1.13(1.04–1.23),p = 0.003)increased susceptibility of IBS and higher levels of stearate(18:0)(OR(95%CI):0.72(0.58–0.89),p = 0.003) decreased susceptibility of IBS.

**Conclusion:**

The metabolites implicated in the pathogenesis of IBS possess potential as biomarkers and hold promise for elucidating the underlying biological mechanisms of this condition.

## Introduction

Irritable bowel syndrome (IBS) is a functional gastrointestinal disorder that significantly affects individuals’ quality of life and social functioning. Most European countries, the United States, and China report prevalence rates ranging from 5% to 10% [1]. Clinical symptoms of IBS include abdominal pain or discomfort, stool irregularities, bloating, as well as other somatic, visceral, and psychiatric comorbidities [2]. Current therapeutic approaches for IBS primarily focus on alleviating symptoms and are often limited in their effectiveness [3]. Due to the chronic nature of IBS, patients with this condition impose a significant economic burden on healthcare systems. In particular, the foundation for both disease prevention and treatment is the investigation of the biological process. However, the underlying mechanisms of IBS remain poorly understood, encompassing a range of characteristics including gastrointestinal motility, visceral hypersensitivity, intestinal secretion and intestinal permeability [2, 4]. It is important to acknowledge that genome-wide association studies (GWAS) have identified numerous genetic variations that enhance the vulnerability of individuals to IBS, thereby offering molecular insights into the complex interplay between environmental and genetic factors in the pathogenesis of IBS [5, 6].

Advancements in microbiological technologies, such as metagenomics, metabolomics have shed light on microbial functions, metabolites, and their interactions with the host, which hold promise in unraveling the biological processes underlying the disease [7]. Given their involvement in processes like cellular organization, post-translational modification, and epigenetic modification, it is essential to further explore the potential links between metabolic profiling and disease risks [8]. In addition, an increasing number of evidence has demonstrated that an altered metabolic profile in either the host or the gut microbiota, as well as their interactions, likely play a significant role in the manifestation of IBS symptoms [9–11].

In recent times, the utilization of Mendelian randomization (MR) analysis has been increasingly prominent as a valuable method within the field of epidemiological research. Mendelian randomization (MR) employs genetic variants as instrumental variables (IVs) to establish causal links between exposures and outcomes. Compared to other epidemiological approaches, MR provides unbiased estimates by leveraging genotypes determined at conception, making it less susceptible to confounding factors and reverse causation [12]. Currently, although some evidence shows the role of blood metabolites in IBS, a causal relationship has not been established. Hence, in order to acquire a more profound comprehension of the development of IBS, MR is utilized as a potent instrument to explore the plausible causal association between metabolites and IBS.

## Materials and methods

### Study design

**Fig 1:**
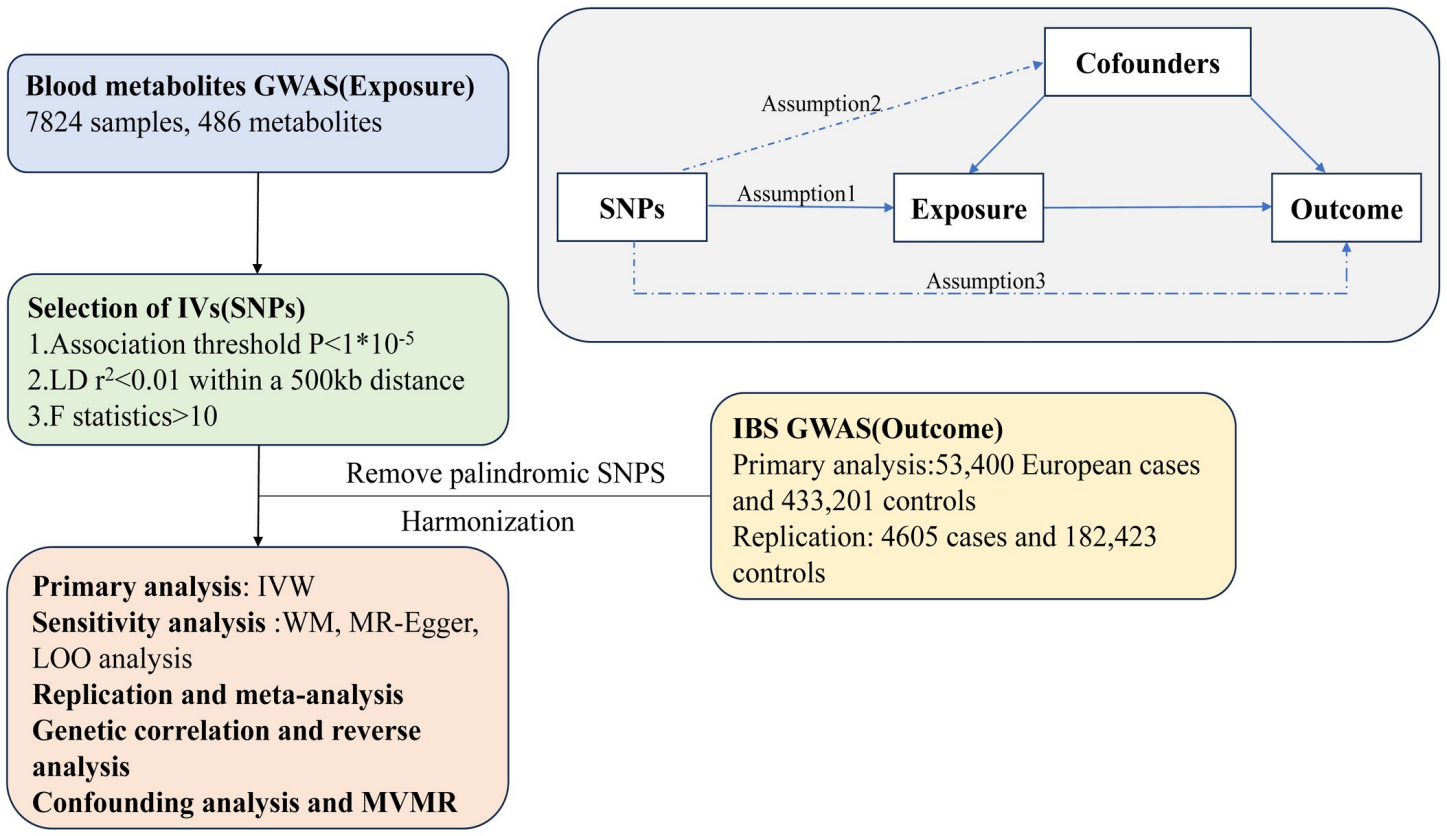
An overview of this Mendelian randomization analysis. IVW, inverse variance weighted; WM, weighted median; LOO analysis, leave-one-out analysis; MVMR, multivariable Mendelian randomization analysis; SNPs, single nucleotide polymorphisms.

A set of single-nucleotide polymorphisms (SNPs) that indicate genetic variations were chosen as instrumental variables for a two-sample Mendelian randomization (MR) analysis. The following three basic theories were accepted (Fig 1): 1. instrumental variables are directly associated with the exposure; 2. instrumental variables are independent of any confounding factors; 3. genetic variants influence outcomes only through the exposure. Fig 1 shows an overview of the study design. The study utilized MR analysis to examine the causal links that exist in both directions between metabolites and IBS [13].

### Data sources

Two GWASs of IBS were used. The discovery set encompassed 53,400 European cases and 433,201 European controls, while the validation set comprised a total of 4,605 cases and 182,423 controls(finn-b-K11_IBS) [6]. At least one of the following four requirements should be met by the IBS cases from UKB [14]: Rome III symptom criteria for the digestive health questionnaire (DHQ) are met if the symptoms of IBS cannot be adequately explained by another diagnosis [15]; DHQ “self-report”: responded positively when asked if they had ever received an IBS diagnosis [16]; Unprompted ‘self-report”: spontaneously declared an IBS diagnosis when asked if they were told of having any serious medical conditions [17]; the International Code of Disease Version 10 (ICD-10). The diagnosis from FinnGen consortium conforms to the ICD-10 criteria.

The metabolomics GWAS server (http://metabolomics.helmholtz-muenchen.de/gwas/) provided the genetic association data for serum metabolites. The ground-breaking research by Shin et al. [18], who conducted the most thorough investigation of genetic impacts on human metabolism to date, was noteworthy. 486 metabolites with genetic effects on human serum metabolites were effectively found by their comprehensive GWAS research. A total of 7,824 people from two European demographic cohorts were included in the study. Local committees approved both trials for ethical purposes, and before participating in the research, each subject gave their voluntarily informed consent. Following stringent quality control procedures, a total of 486 metabolites were analyzed, comprising 309 known metabolites and 177 unknown metabolites [19].

### Selection of genetic instruments

First, a more stringent threshold (p <1×10^−5^) was established as the genome-wide significant cutoff to identify highly correlated SNPs with blood metabolites and IBS, as shown by prior research [20]. In order to mitigate the presence of linkage disequilibrium, we clumped these SNPs (kb = 500, r^2^ = 0.01) which were widely used in previous studies. In order to evaluate the robustness of the instrumental variables (IVs), we calculated the F-statistics. In accordance with a standard criterion, IVs exhibiting an F-statistic value of less than 10 were deemed to be weak and consequently eliminated from subsequent analysis [21]. Additionally, we carried out a harmonization process to ensure the alignment of alleles between exposure- and outcome-associated SNPs. This process also involved the removal of palindromic SNPs and SNPs with incompatible alleles.

### Primary analysis and sensitivity analysis

For the primary analysis, we employed inverse-variance weighted (IVW) regression, which was a reliable method for combining effect estimates from numerous SNPs when pleiotropy was balanced [22]. Given the challenges in verifying the "exclusion-restriction" premise of IVW, various sensitivity analyses were conducted including the Weighted Median Estimator and the MR-Egger Regression model. The method of the weighted median estimator was utilized to investigate the impacts of all available SNPs when half the IVs were valid. This approach yielded unbiased estimates of effects [23]. The MR-Egger regression model yielded a relatively robust estimate that was not influenced by the validity of IVs. Additionally, the model accounted for potential horizontal pleiotropy by incorporating the regression slope and intercept [23]. The MR-PRESSO (MR pleiotropy residual sum and outlier) method was employed to assess the presence of potential outliers among instrumental variables (IVs) by utilizing both global and SNP-specific observed residual sum of squares. Outliers with a p-value< 0.05 in the subsequent distortion test were excluded to get a corrected causal effect. After the identification of outliers by MR-PRESSO, MR analyses were performed subsequent to the elimination of these outliers. Furthermore, directional pleiotropy was evaluated on the intercept derived from the MR-Egger regression model [24]. The Q-test was employed to identify any potential violations of the assumption of heterogeneity in the association between individual IVs for the IVW and MR-Egger methods [25]. In order to assess the robustness of the findings, a leave-one-out (LOO) analysis was performed to examine the potential influence of specific SNP on the outcomes. This involved systematically removing each SNP one at a time and subsequently conducting MR analysis to assess their influence. In summary, we meticulously assessed blood metabolites that could potentially have a causal impact on IBS using a multifaceted approach: (1) Significance in the primary analysis, with a p-value below 0.05 derived from IVW. (2) Consistency in both direction and the effect size across three MR methods including IVW, Weighted Median and MR-Egger. (3) The lack of heterogeneity or horizontal pleiotropy in Mendelian randomization (MR) outcomes. (4) the estimates obtained are not severely disturbed by the presence of a single SNP [26]. All MR analyses were performed using the “TwoSampleMR” package (version 0.4.22). The meta-analysis was performed using the Reviewer Manager software (Version 5.4.1). The presentation of causal estimates is in the form of odds ratios (ORs) accompanied by 95% confidence intervals (CIs).

### Replication and meta-analysis

In order to thoroughly assess the reliability of the candidate metabolites identified, we conducted a replication of IVW analysis in an independent IBS cohort consisting of 4,605 cases and 182,423 controls. All cases encompassed within the FinnGen dataset were in accordance with the ICD-10 standard. The FinnGen GWAS incorporated adjustments for many factors, including sex, age, the first 10 genetic main components, genotyping batch, and the genetic relatedness matrix [27].

### Reverse causality analysis and genetic correlation

To rule out the possible bi-directional association between metabolites and IBS, we conducted reverse MR analysis regarding IBS and identified metabolites. The instrumental variables used in this study were chosen based on a genome-wide significance threshold of 5e-6.

In addition, prior research has raised concerns about potential false positives in MR results stemming from genetic correlations between traits [28]. The estimation of coinheritance between two traits based on SNPs can be accomplished using Linkage Disequilibrium Score (LDSC) regression [29]. Therefore, in order to mitigate any confounding due to the genetic link between the screened metabolites and IBS, we employed LDSC to examine the causative effects.

### Confounding analysis and multivariable MR analysis

In addition, we conducted an examination of the IVs for metabolites using the Phenoscanner V2 website (http://www.phenoscanner.medschl.cam.ac.uk/) in order to assess the potential association between each SNP and confounding factors relevant to IBS. If any SNP was identified to exhibit an association with the potential confounding factors at a significance level of p<1×10^−5^, a subsequent MR analysis would be conducted following the exclusion of these SNPs. This step aims to ensure the dependability and validity of the obtained findings.

The utilization of Multivariable Mendelian Randomization (MVMR) enables the adjustment for interactions arising from genetic diversity between exposures, by combining numerous exposures that have the potential to interact with one another. The multivariable mediation analysis examined the specific impact of each exposure variable on the outcome variable, independent of any other exposure variables. Hence, we conducted MVMR analysis on the identified metabolites in order to account for their potential interactions. This adjustment was carried out using the IVW method [30, 31].

## Results

### Causal effects of the blood metabolites on IBS

The number of instrumental variables of metabolite varied from 3 to 483, with a median number of 20.7. The IVs have the potential to account for a range of 0.24% to 70.8% of the variance in their associated metabolites. All of the minimal F statistics exceeded a value of 10, suggesting a potential presence of a weak instrumental variable bias. Details of the SNPs can be seen in S3 Table.

**Fig 2:**
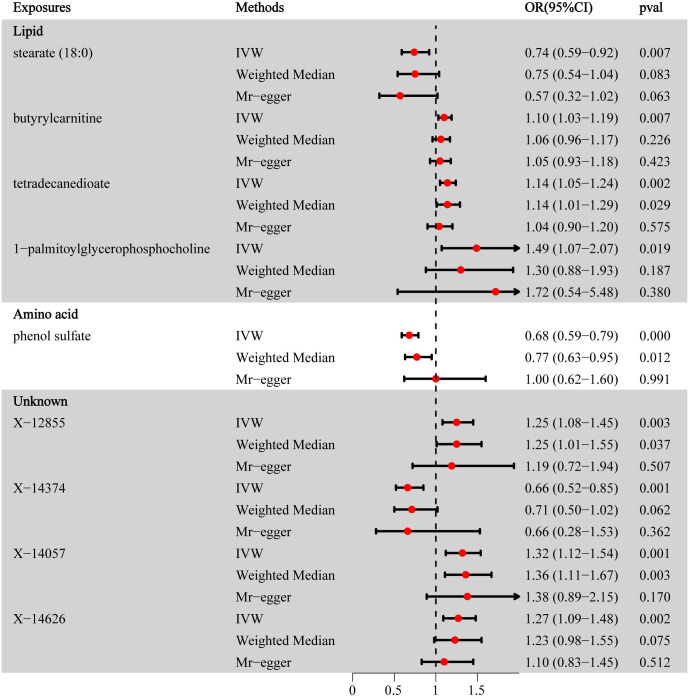
Forest plot for the causality of metabolites on IBS using different analysis. OR, odds ratio;95% CI, 95% confidence interval.

The IVW MR analysis was conducted for each pair of metabolites, utilizing the instrumental variables available. A collective sum of 41 statistically significant correlations (P<0.05) were detected, comprising 25 previously recognized metabolites and 16 metabolites that have not been previously characterized. In addition, sensitivity and pleiotropy studies were performed to assess the durability of the observed correlations (Table 1). There was no observed indication of heterogeneity, as shown by a Cochran Q test with a p-value greater than 0.05. Additionally, both the MR-Egger regression and MR-PRESSO global test did not reveal any pleiotropic effects (p>0.05). We further used a strict selection mentioned above and finally, 9 metabolites were included. The direction and magnitude were consistent in other sensitivity analyses, although p-value was not always significant. (Fig 2) The findings of the study indicate that there is a negative correlation between levels of stearate (18:0) (OR(95%CI): 0.74 (0.59–0.92), p = 0.007), phenol sulfate (0.68 (0.59–0.79), p = 0.000)and IBS. Elevated levels of butyryl carnitine (OR(95%CI): 1.10 (1.03–1.19), p = 0.007), tetradecanedioate (OR(95%CI): 1.14 (1.05–1.24), p = 0.002), and 1-palmitoylglycerophosphocholine (OR(95%CI): 1.49 (1.07–2.07), p = 0.019) were found to be significantly correlated with a heightened susceptibility to IBS. Three unknown metabolites may also be risk factors for IBS except X-14374. More Details can be seen in S2 Table.

**Table 1:** MR analyses of genetically predicted levels of blood metabolites and risk of IBS.

Exposures	nsnp	OR(95%CI)	pval	Het.pval	MR-egger intercept pval	MR-presso global test pval
**Amino acid**						
methionine	22	1.66(1.02–2.71)	0.043	0.522	0.514	0.547
arginine	21	1.36(1.07–1.74)	0.012	0.445	0.462	0.455
valine	6	0.53(0.29–0.95)	0.034	0.863	0.780	0.830
serotonin (5HT)	16	1.25(1.04–1.50)	0.020	0.683	0.295	0.684
homocitrulline	7	1.26(1.01–1.57)	0.043	0.908	0.842	0.909
N-acetylglycine	16	1.11(1.01–1.23)	0.033	0.797	0.399	0.812
trans-4-hydroxyproline	6	0.79(0.63–1.00)	0.050	0.521	0.298	0.618
phenol sulfate	15	0.68(0.59–0.79)	0.000	0.225	0.122	0.304
Isovalerylcarnitine*	19	0.83(0.71–0.98)	0.031	0.232	0.119	0.007
indolelactate	19	0.79(0.64–0.98)	0.030	0.733	0.592	0.765
**Lipid**						
1-palmitoylglycerol (1-monopalmitin)	14	1.49(1.07–2.07)	0.019	0.068	0.805	0.083
stearate (18:0)	43	0.74(0.59–0.92)	0.007	0.630	0.352	0.677
glycocholate	9	0.90(0.82–0.99)	0.027	0.999	0.903	1.000
Butyryl carnitine	33	1.10(1.03–1.19)	0.007	0.277	0.287	0.296
docosapentaenoate (n3 DPA)*	10	1.25(1.01–1.55)	0.041	0.365	0.448	0.032
1-palmitoylglycerophosphocholine	34	0.58(0.43–0.78)	0.000	0.258	0.353	0.292
10-nonadecenoate (19:1n9)	7	0.71(0.54–0.92)	0.011	0.482	0.506	0.514
1-arachidonoylglycerophosphoinositol*	20	1.24(1.02–1.52)	0.033	0.182	0.222	<0.001
1-palmitoylglycerophosphoinositol	11	0.84(0.71–0.99)	0.034	0.794	0.488	0.752
tetradecanedioate	21	1.14(1.05–1.24)	0.002	0.868	0.145	0.879
hexadecanedioate	24	1.13(1.02–1.25)	0.024	0.725	0.904	0.766
4-androsten-3beta,17beta-diol disulfate 2	19	1.19(1.01–1.40)	0.040	0.150	0.220	0.148
**Carbohydrate**						
1,5-anhydroglucitol	31	0.83(0.69–0.99)	0.044	0.126	0.903	0.153
**Xenobiotics**						
Paraxanthine*	12	1.14(1.02–1.26)	0.020	0.717	0.182	0.013
**Peptide**						
aspartylphenylalanine	7	1.21(1.02–1.43)	0.029	0.936	0.687	0.93
**Unknown**						
X-09706	22	0.81(0.65–1.00)	0.047	0.589	0.341	0.607
X-11437	15	0.93(0.87–1.00)	0.037	0.896	0.467	0.910
X-11438	24	0.85(0.73–0.99)	0.036	0.070	0.496	0.088
X-11521	12	0.83(0.69–0.99)	0.037	0.779	0.356	0.780
X-11537	6	0.78(0.63–0.96)	0.021	0.171	0.932	0.248
X-12038*	57	1.35(1.06–1.73)	0.015	0.025	0.854	0.002
X-12063	17	1.09(1.00–1.19)	0.047	0.128	0.313	0.190
X-12212	11	1.16(1.03–1.29)	0.010	0.584	0.219	0.663
X-12230*	8	0.77(0.61–0.99)	0.041	0.058	0.470	0.015
X-12855	20	1.25(1.08–1.45)	0.003	0.644	0.826	0.538
X-13435*	25	0.78(0.64–0.94)	0.011	0.048	0.005	0.043
X-13477	8	0.70(0.50–0.97)	0.031	0.605	0.586	0.631
X-13741	12	0.87(0.77–0.97)	0.016	0.281	0.321	0.345
X-14057	18	1.32(1.12–1.54)	0.001	0.114	0.817	0.143
X-14374	9	0.66(0.52–0.85)	0.001	0.376	0.975	0.372
X-14626	18	1.27(1.09–1.48)	0.002	0.859	0.232	0.889

Note:

*Outliers detected by MR-PRESSO were removed.

SNP, single nucleotide polymorphism; OR, odds ratio; CI, confidence interval; Het.pval, heterogeneity test p value.

### Replication and meta-analysis

**Fig 3:**
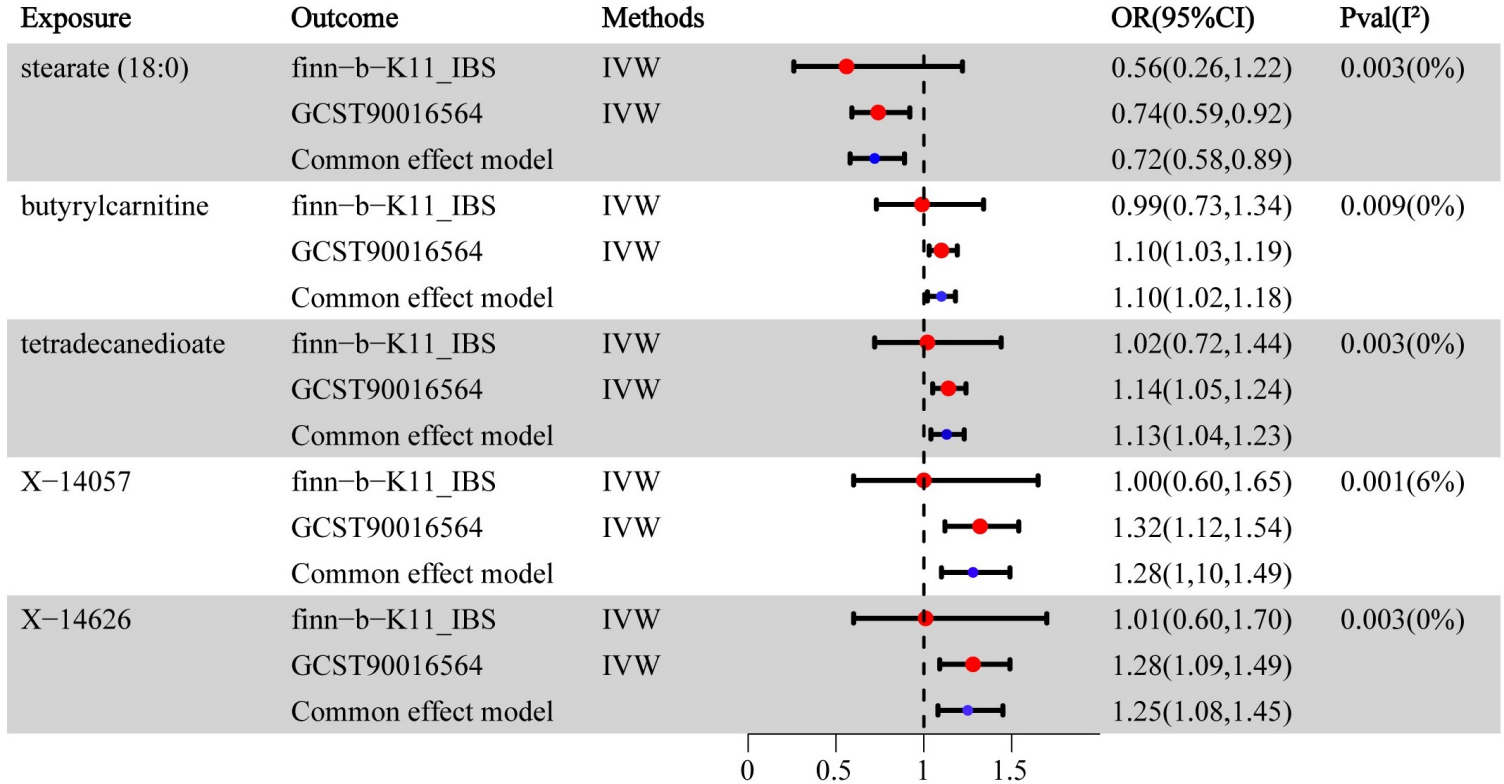
Meta-analysis of the significant association between metabolites and IBS. OR, odds ratio;95% CI, 95% confidence interval.

**Fig 4:**
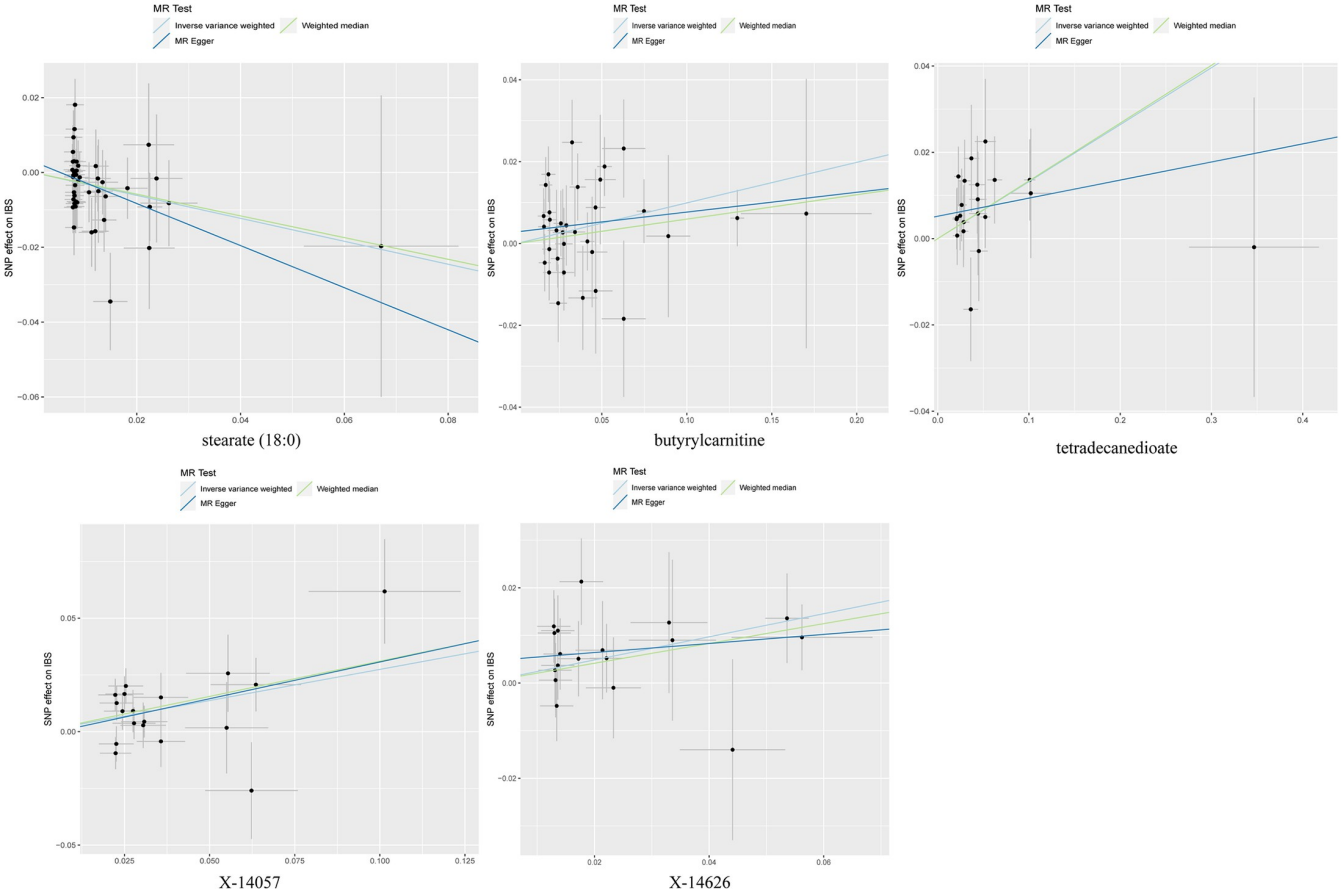
Scatterplot for the significant MR association between metabolites and IBS. SNP, single nucleotide polymorphism.

Additionally, we performed a MR analysis using a separate dataset from the FinnGen GWAS, which consisted of 4,605 cases and 182,423 controls. The findings of the meta-analysis have established that there exists a relationship between five specific metabolites and their impact on IBS (Fig 3). Genetically increased levels of butyryl carnitine(OR(95%CI):1.10(1.02–1.18),p = 0.009), tetradecanedioate(OR(95%CI):1.13(1.04–1.23),p = 0.003)increased the susceptibility of IBS and higher levels of stearate(18:0)(OR(95%CI):0.72(0.58–0.89),p = 0.003) decreased the susceptibility of IBS. The scatterplots of selected metabolites are shown in Fig 4.

### Reverse association analysis and genetic correlation analysis

We conducted a MR analysis employing instrumental variables for IBS in order to determine the reverse casual effects of IBS on metabolites. Since limited SNPs can be obtained when clumping, we extended the limitation of p-value to 5e-6. The findings did not demonstrate a reverse causal association with IBS. Furthermore, no evidence substantiating horizontal pleiotropy was found. More details can be found in S4 Table.

In order to explore an alternate hypothesis about the presence of similar genetic components, we used LDSC analyses to assess the potential influence of shared causative genetic variations on the observed genetic relationships. Regarding LDSC analysis, we did not detect significant genetic connections. (rg = 0.098 and p = 0.084 between and stearate (18:0) and IBS; rg = 0.017 and p = 0.881 between butyryl carnitine and IBS; rg = 0.075 and p = 0.384 between tetradecanedioate and IBS) (S5 Table).

### Cofounding factors and MVMR

We conducted an investigation to determine the potential independence of SNPs linked to five metabolites from potential risk factors using the Phenoscanner and potential SNPs were eliminated from the analysis. The results showed that despite this exclusion, estimates obtained continued to exhibit statistical significance. More details can be seen in S6 Table. After adjusting for metabolite interactions, MVMR estimates showed that genetically predicted stearate (18:0), butyryl carnitine and tetradecanedioate can still influence IBS independently of other metabolites (S7 Table).

## Discussion

IBS presents a complex diagnostic challenge due to the absence of reliable biomarkers or definitive tests. Diagnosis relies primarily on questionnaires, which, though commonly used, struggle to provide quantifiable and objectively reproducible data. Furthermore, the heterogeneous nature of IBS symptoms complicates its identification [2]. Some genetic variants associated with a heightened risk of IBS have been identified, shedding light on the intricate interplay between genetic and environmental factors in IBS development [6]. Prior research has also elucidated diverse alterations in metabolites, including short-chain fatty acids (SCFAs) in IBS. Nevertheless, the precise causal link between these metabolites and IBS remains unknown. Additionally, there remains a scarcity of specific and sensitive diagnostic markers for IBS. This study employed a two-sample MR approach to investigate the causal relationship between 486 blood metabolites and the risk of IBS. An additional GWAS dataset was utilized for the purpose of replication and meta-analysis. The findings indicate that elevated concentrations of butyryl carnitine and tetradecanedioate are associated with heightened vulnerability to IBS, while increasing levels of stearate (18:0) exhibit a protective effect. To the best of our understanding, this work is the initial MR investigation to evaluate the systematic and comprehensive impact of human blood metabolites on IBS. Furthermore, reverse causality was not found and these associations do not appear to be confounded by genetic correlation. MVMR estimates suggested that stearate(18:0), butyryl carnitine and tetradecanedioate can directly affect IBS independently of other metabolites.

Our study showed that stearic (18:0) played a protective role in IBS. Stearic acid is a long-chain fatty acid consisting of 18 carbon atoms. From a dietary perspective, stearic acid is considered relatively neutral when compared to some other saturated fatty acids. It holds a significant role in various bodily processes such as inflammation regulation, coagulation, glucose balance, and bile acid metabolism. One study even noted a significant difference in stearic acid levels between IBS patients and healthy individuals [9]. Additionally, Zhang recently reported that 17 fatty acids, all belonging to the medium-chain and long-chain fatty acid categories (C6 and longer), were downregulated in IBS-D (Diarrhea-Predominant IBS) patients. He suggested that medium-chain and long-chain fatty acids present in the colonic mucosa could serve as potential markers for distinguishing IBS-D patients from healthy subjects. It is essential to conduct further studies to validate these findings and gain a more comprehensive understanding of their significance in IBS [11]. The mechanism underlying it was not clear. A study found that a diet enriched with stearic acid led to reduced concentrations of fecal secondary bile acids (SBA). These SBA can impact intestinal function, including factors like intestinal peristalsis and mucosal permeability, which may have a key role in the mechanism of IBS [32].

Tetradecanedioate, also known as “myristic acid”, is a dicarboxylic acid with a 14-carbon atom chain. It can contribute to energy production in cells and also serve as a precursor for the synthesis of essential lipids and potentially acts as a signaling molecule in cellular processes [33]. Han’s research discovered that individuals with IBS exhibited higher levels of tetradecanoyl-CoA [9]. Tetradecanoyl-CoA represents a specific intermediate in the degradation of tetradecanoic acid. During the process of beta-oxidation, tetradecanoyl-CoA is broken down into smaller acyl-CoA molecules, eventually leading to the production of acetyl-CoA units. Some of these acetyl-CoA units may be further metabolized to generate tetradecanedioate as an intermediate product [33, 34]. Although the studies were limited, the dysregulation of tetradecanedioate might be implicated in the development of IBS.

Butyryl carnitine is a conjugate of butyric acid and carnitine. It is formed as part of the fatty acid metabolism process, specifically involving short-chain fatty acids like butyric acid. The role of butyryl carnitine in the body is primarily related to the transport and utilization of short-chain fatty acids as an energy source [35]. Short-chain fatty acids, including butyric acid, could enhance the gut epithelial barrier and accelerate the repair of it [36]. The benefits of butyric acid are still controversial. While some reports suggest it may be a potential protective factor for IBS, other studies present opposing views. For instance, Tian reported an increase in butyric acid levels in the serum of patients with IBS-D (Diarrhea-Predominant IBS) but not in their feces. Jakobsdottir [37] pointed out that fecal short-chain fatty acids may not necessarily reflect the levels in the colon and that blood analysis might offer a more accurate alternative. It’s important to note that while moderate levels of short-chain fatty acids may contribute to stabilizing intestinal permeability by directly influencing the distribution of tight junction proteins, high concentrations of these fatty acids may have the opposite effect [38, 39]. Excessive activation of intestinal immunity is one potential mechanism through which short-chain fatty acids might play a role in the pathogenesis of IBS [40]. The effect of butyryl carnitine on IBS patients is still not fully understood. Both butyryl carnitine and butyric acid are involved in the metabolic processes of fatty acids which may have an impact on the underlying mechanisms of IBS pathology. Nevertheless, further research is needed [41].

Several limitations should be noted in our study. First, the selected metabolites did not achieve statistical significance following stringent Bonferroni or False Discovery Rate (FDR) corrections. Consequently, we can only suggest these metabolites as potentially associated with IBS. Secondly, given the heterogeneous nature of IBS, encompassing multiple subtypes (IBS-D, IBS-C, IBS-M), the relationship between metabolism and genetics may vary among individuals and subtypes [42]. Thirdly, our study did not provide evidence supporting a connection between butyryl carnitine and IBS, indicating that this relationship remains theoretical. Furthermore, there have been limited investigations into serum metabolism in patients with IBS, potentially due to the lack of appropriate analytical methods. In the present investigation, our assessment was limited to examining the impact of metabolites detected in blood on IBS, without taking into account metabolite concentrations in tissues that are more biologically pertinent. Hence, additional validation and exploration are necessary to substantiate our conclusions.

In summary, our MR analysis indicates that stearic acid, tetradecanedioate, and butyryl carnitine may serve as potential metabolite markers in the context of IBS. The metabolites implicated in the pathogenesis of IBS have potential as biomarkers and can contribute to the elucidation of the underlying biological mechanisms of this condition.

## Supporting information

S1 Table Summary information for the genetic data used in the present study. (XLSX)

S2 Table MR analysis of all metabolites identified in MR analysis. (XLSX)

S3 Table Harmonization data of selected blood metabolites and IBS. (XLSX)

S4 Table Reverse MR analysis. (XLSX)

S5 Table The SNP-based heritability (h^2^) of the metabolites. (XLSX)

S6 Table Confounders identified from phenoscanner. (XLSX)

S7 Table Multivariable MR analysis of the identified metabolites. (XLSX)

S1 File Supplementary materials. (DOCX)
