# Supplementary material for: Causality of genetically determined blood metabolites on irritable bowel syndrome: A Mendelian randomization study
Source: PLoS One. 2024 Apr 3;19(4):e0298963. doi: 10.1371/journal.pone.0298963 (PMC10990233; doi:10.1371/journal.pone.0298963)

Supplementary Material

**Causality of genetically determined blood metabolites on irritable bowel syndrome: A Mendelian Randomization Study**

**Supplementary Figures**

**S1 Fig:** **Forest plots for the Mendelian randomization (MR) leave-one-out analysis of the significant inverse variance weighted (IVW) estimates.**


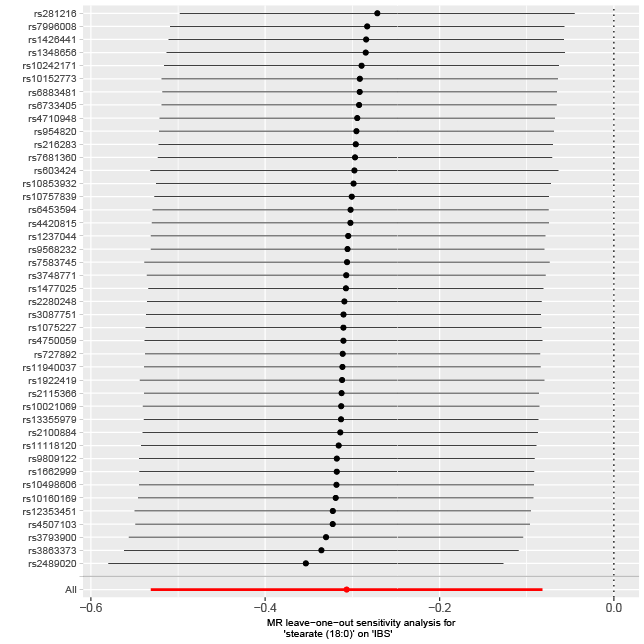


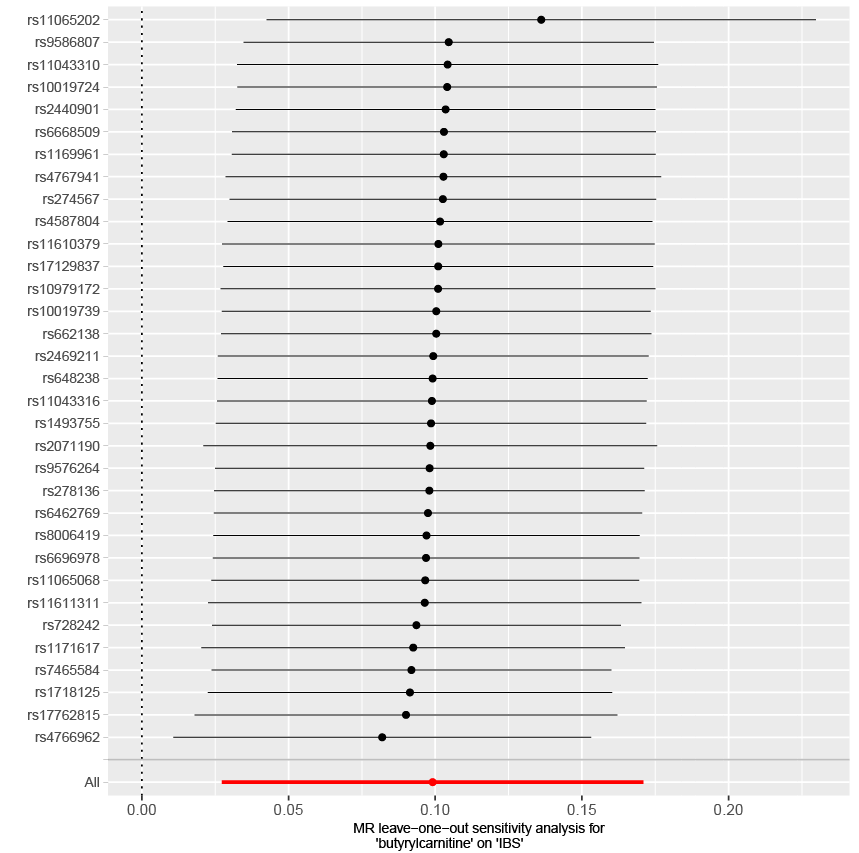


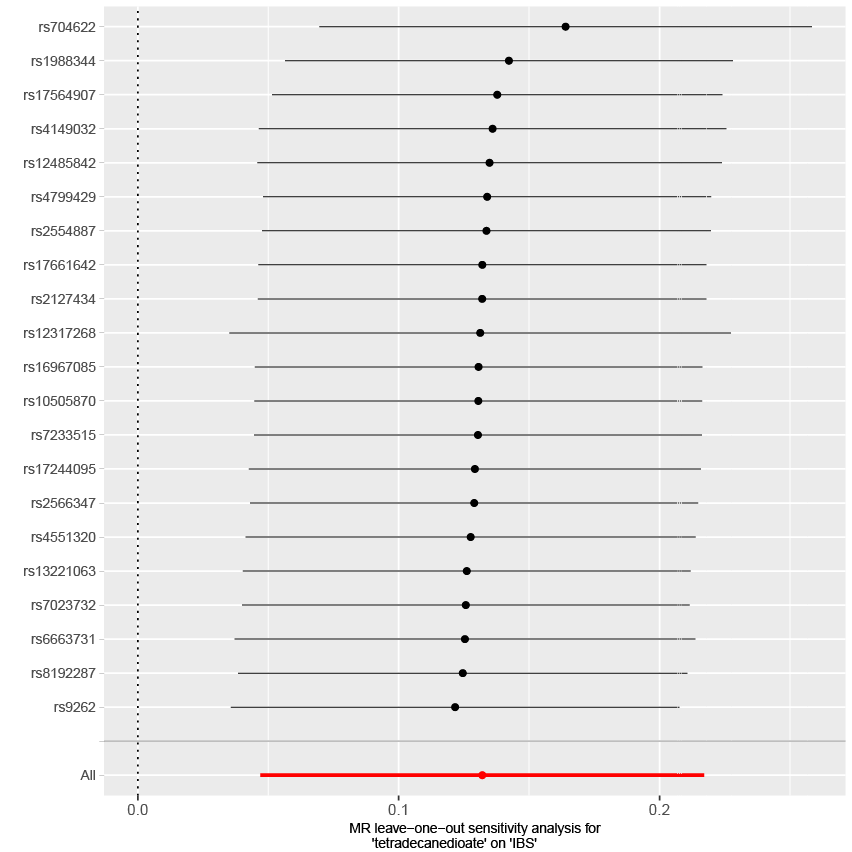


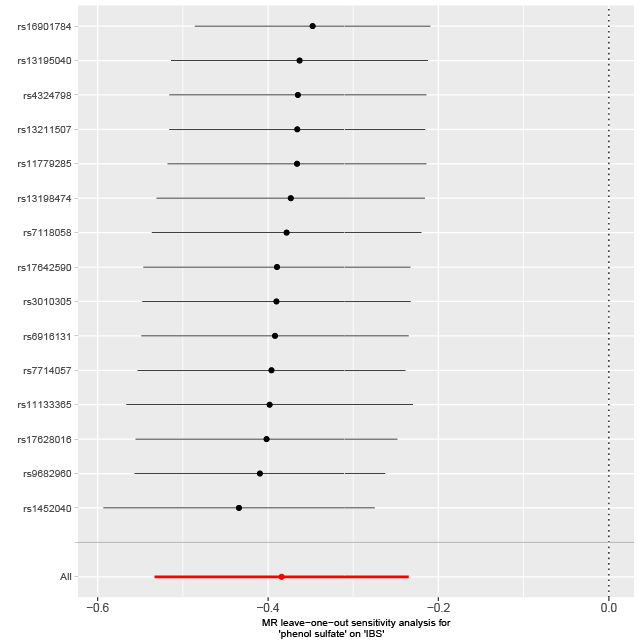


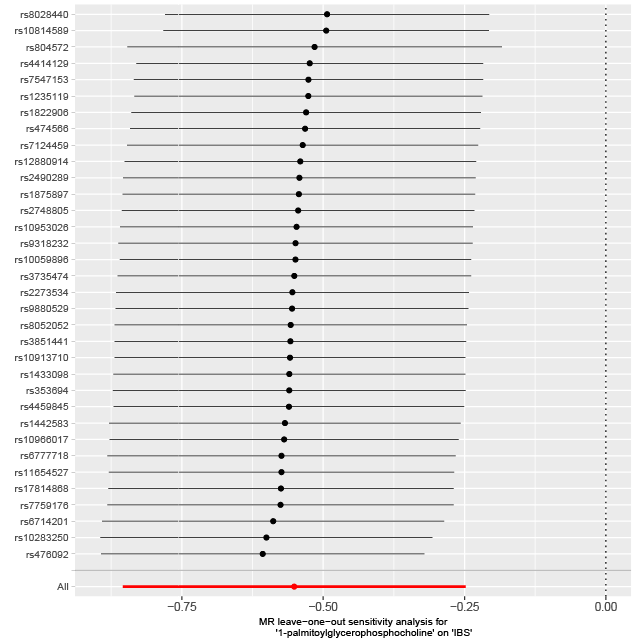


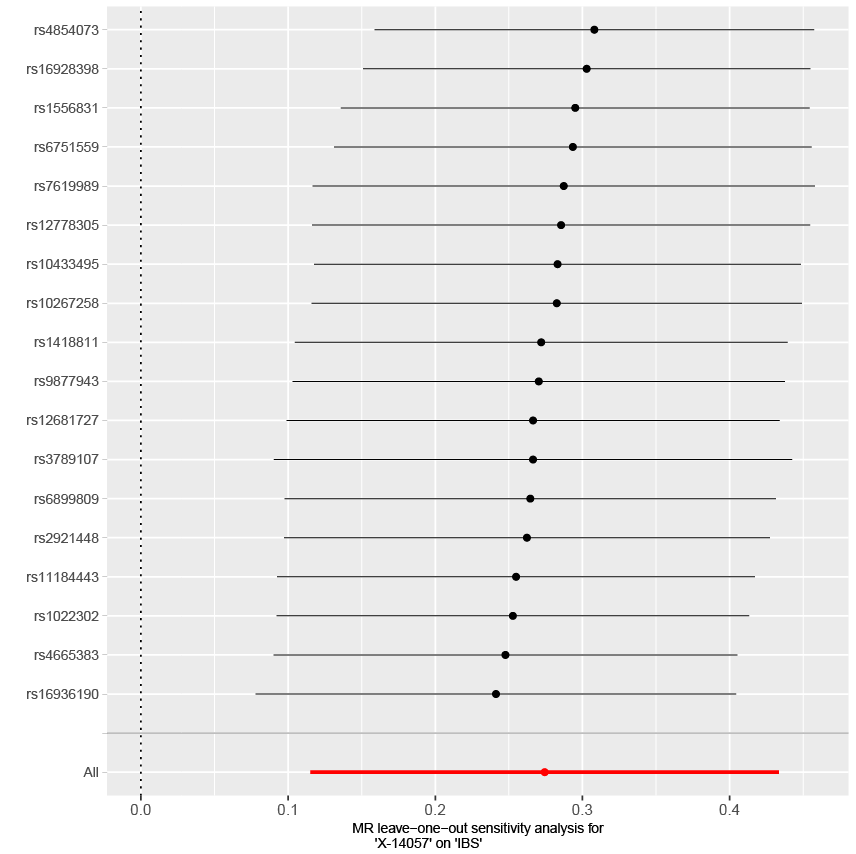


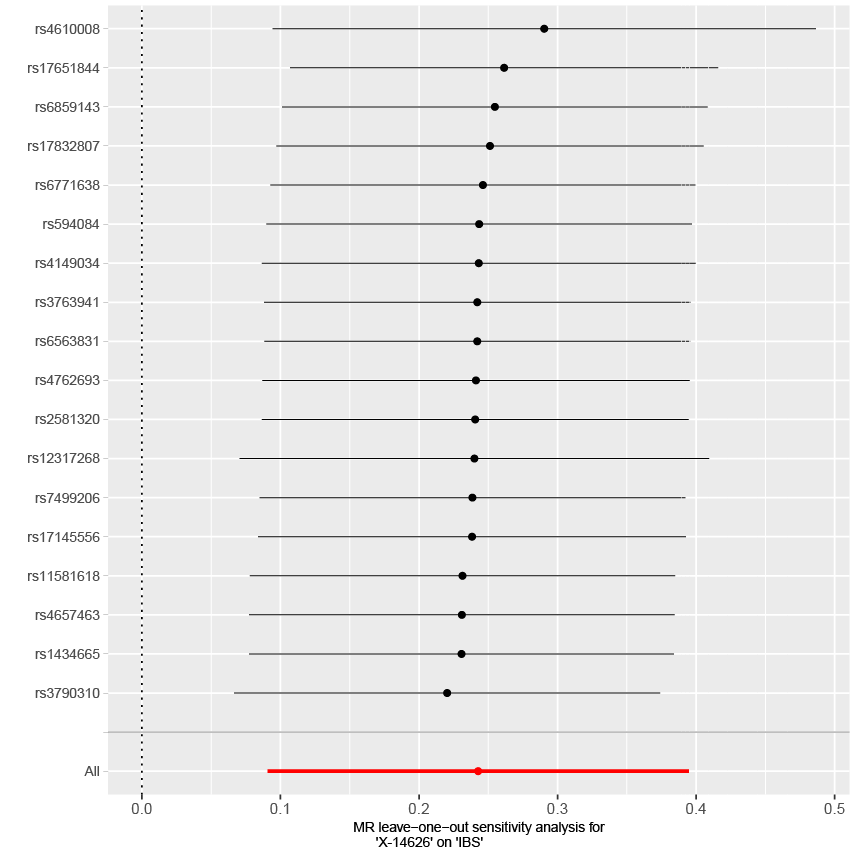


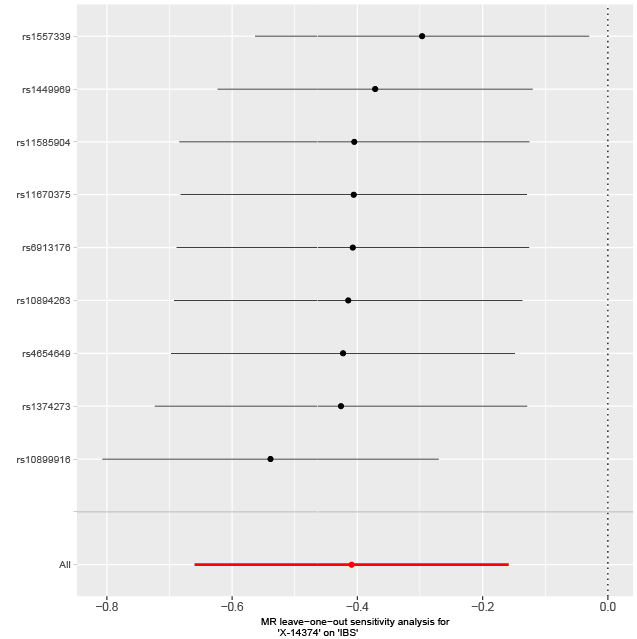


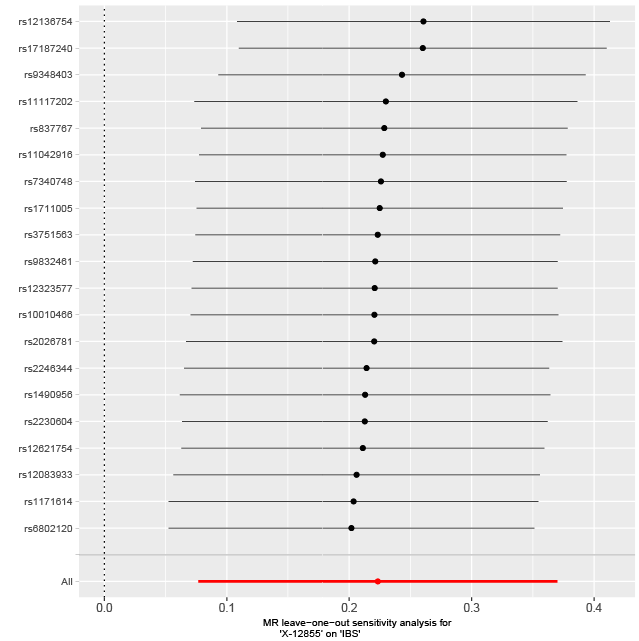


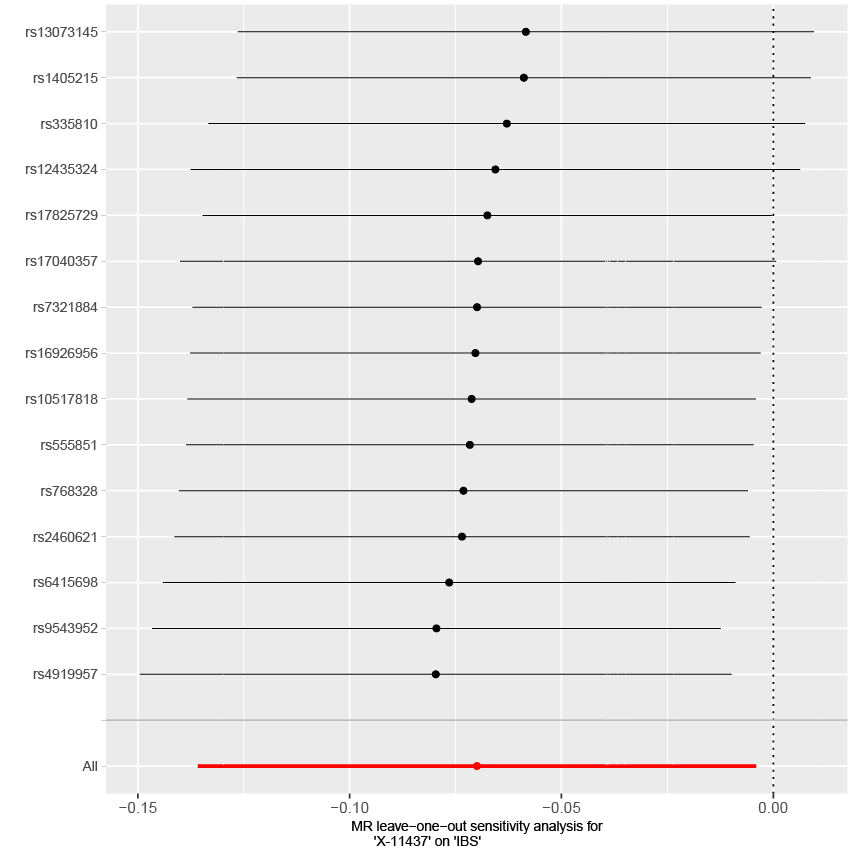


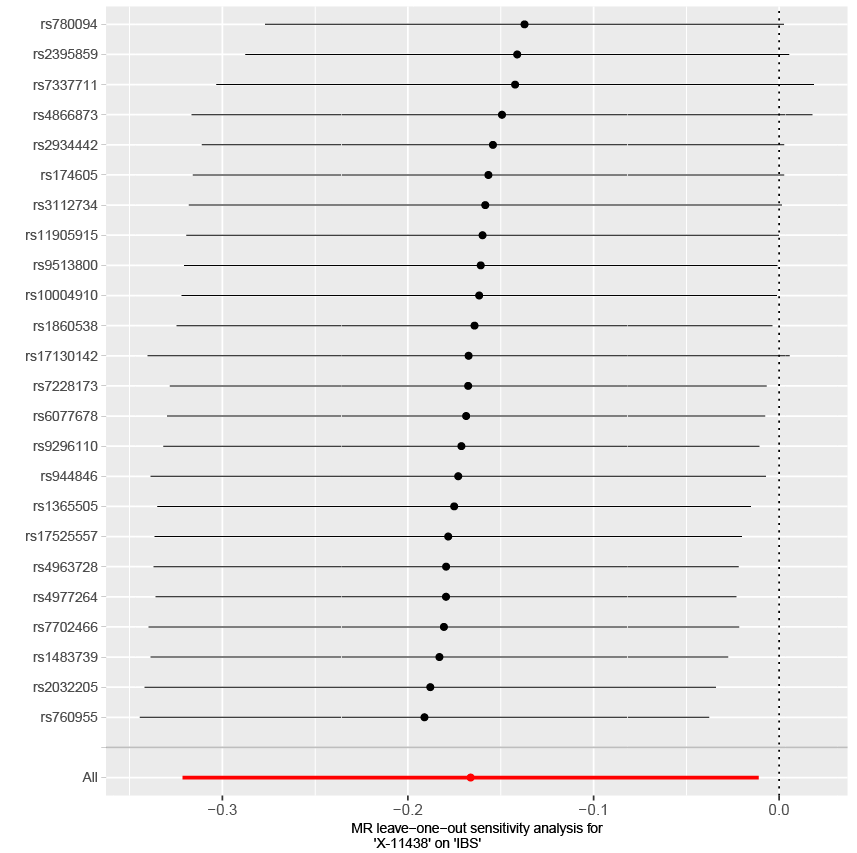


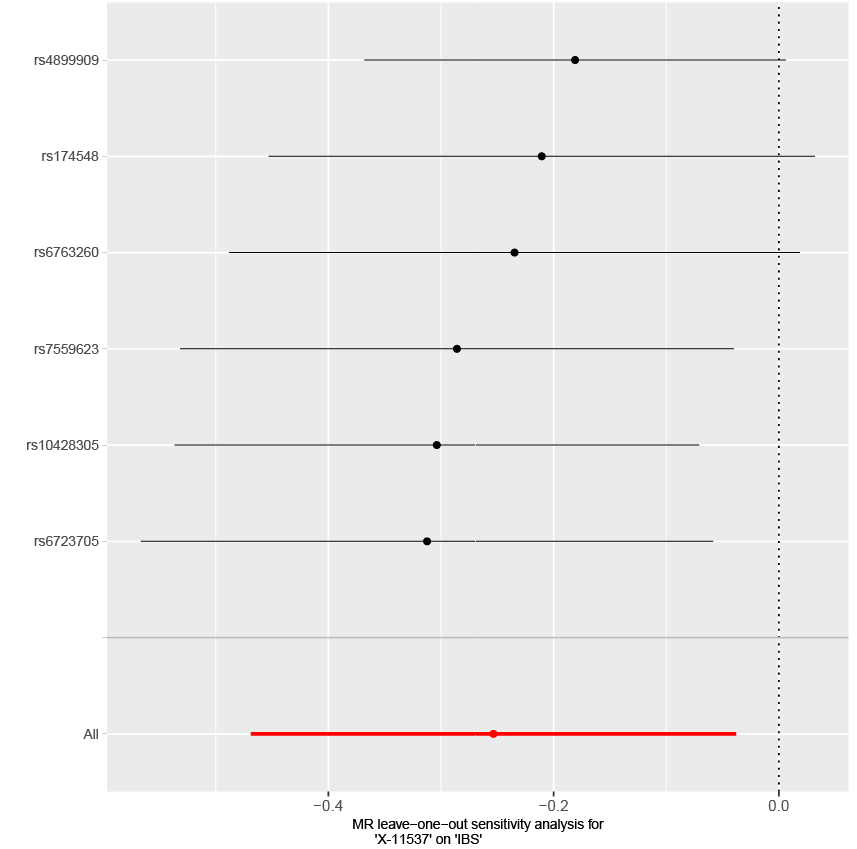


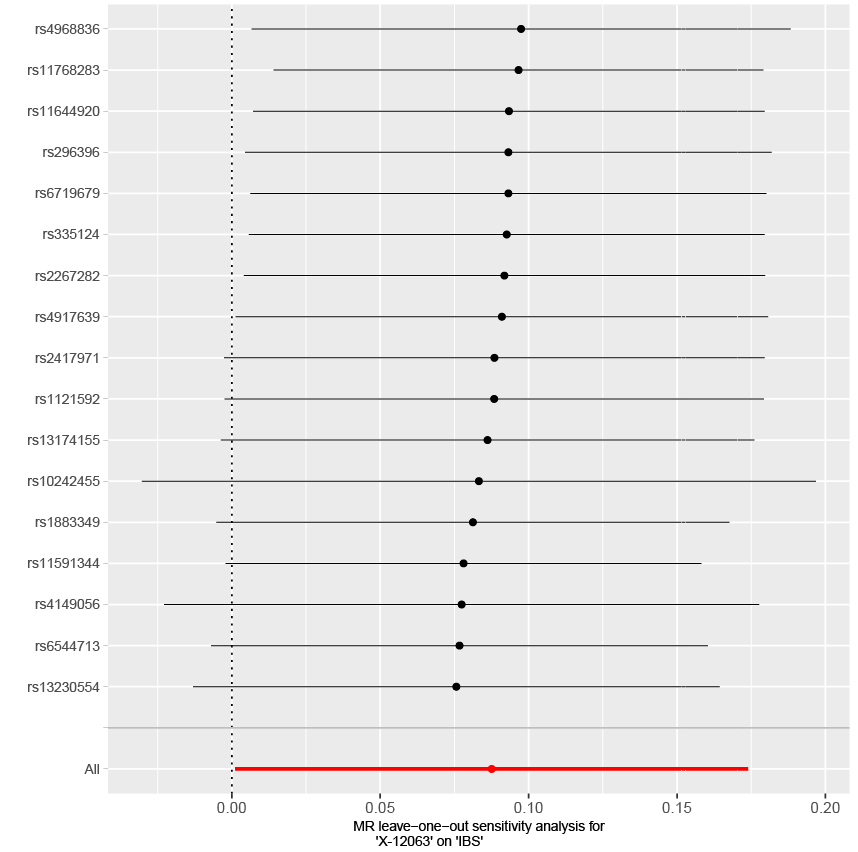


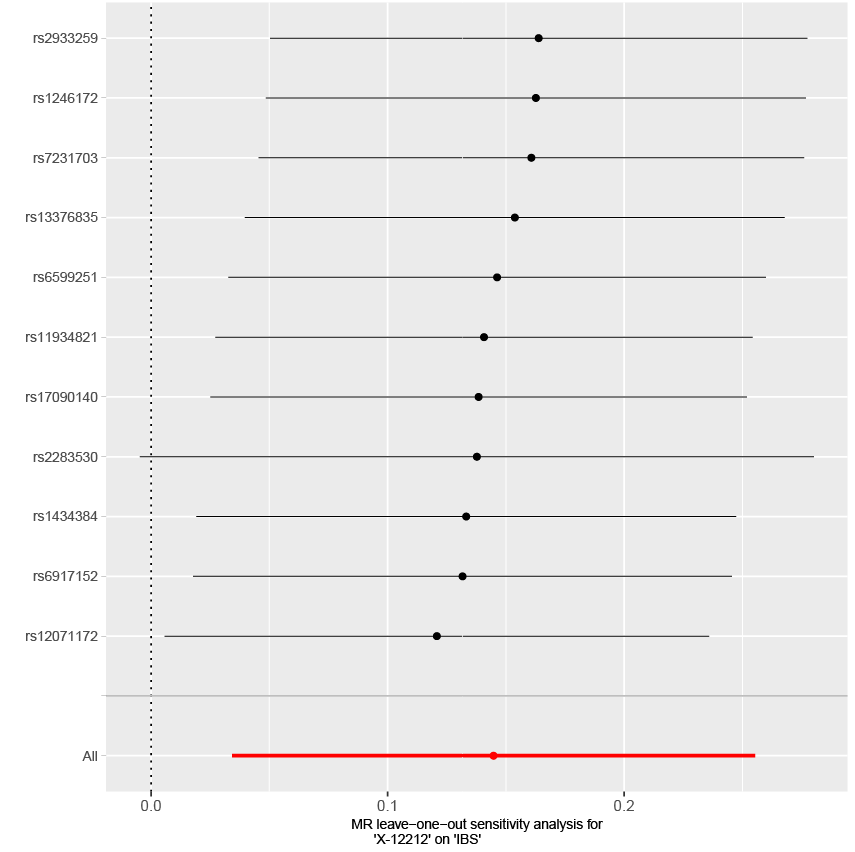


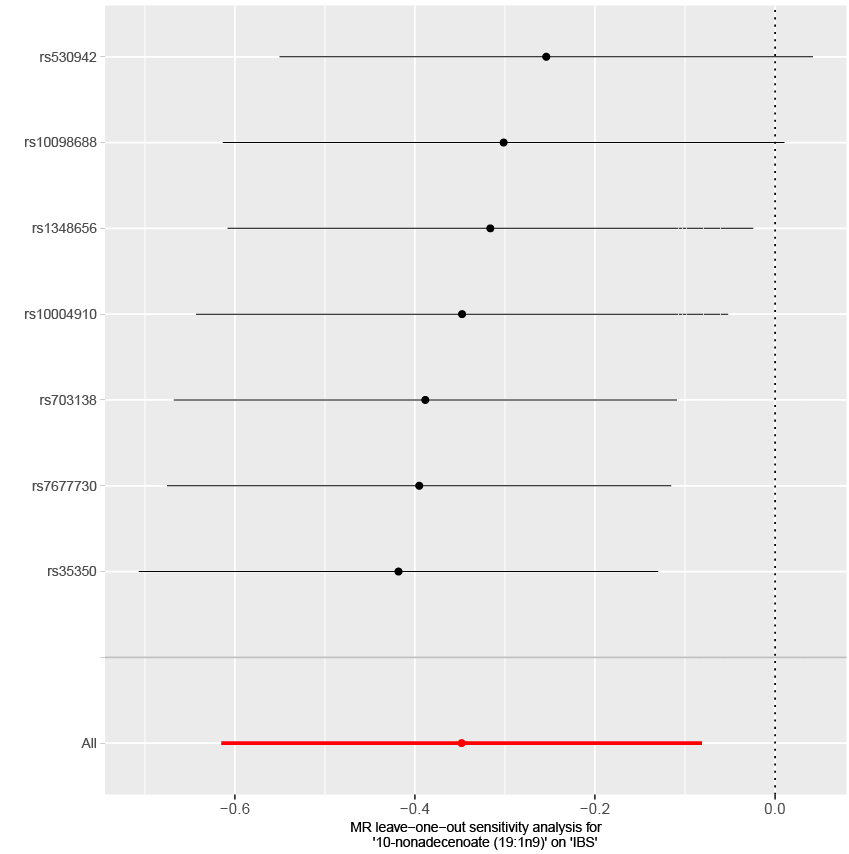


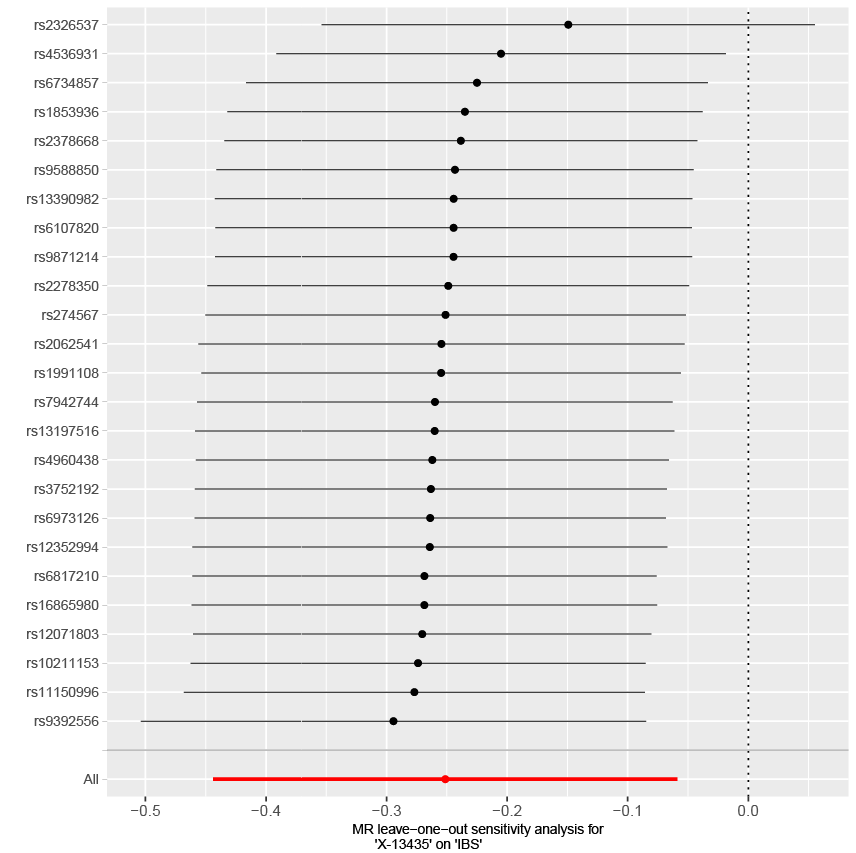


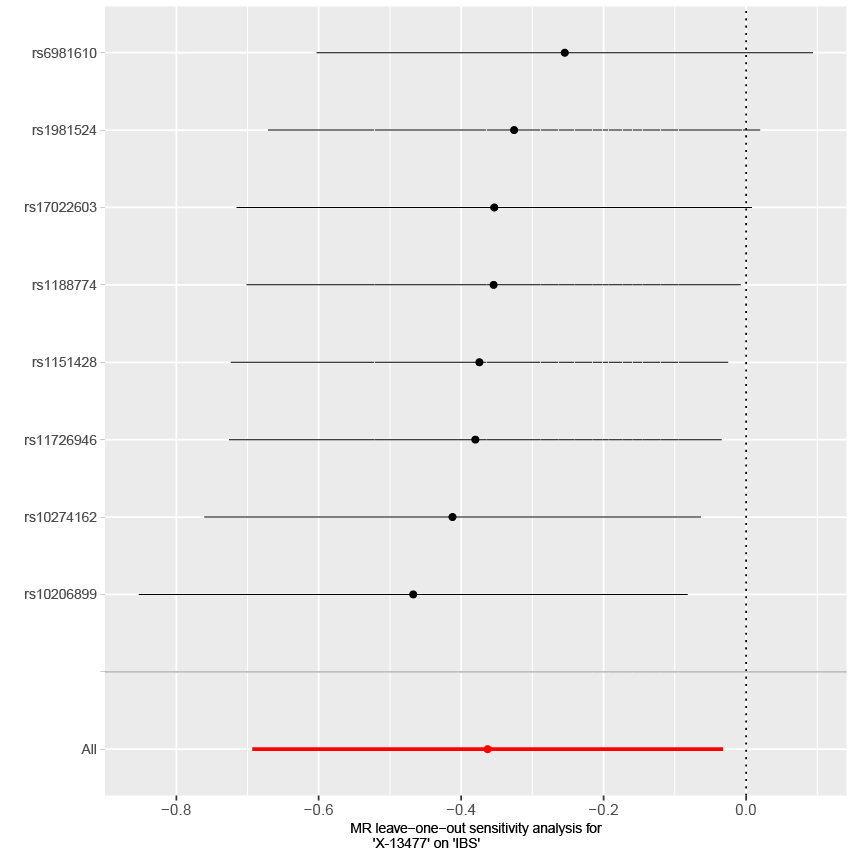

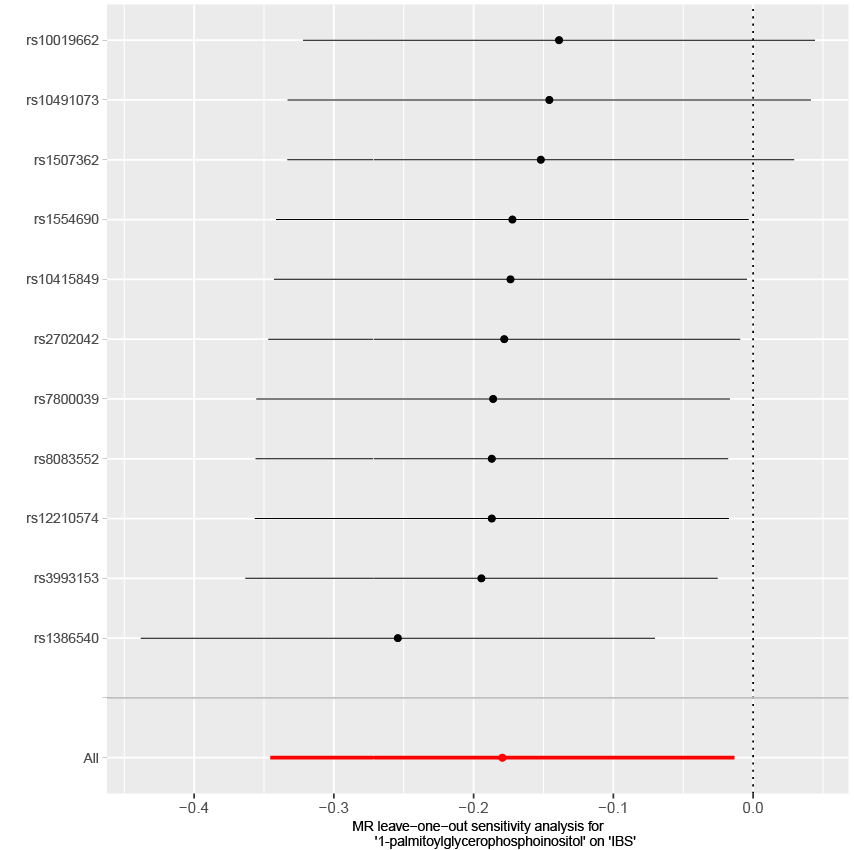

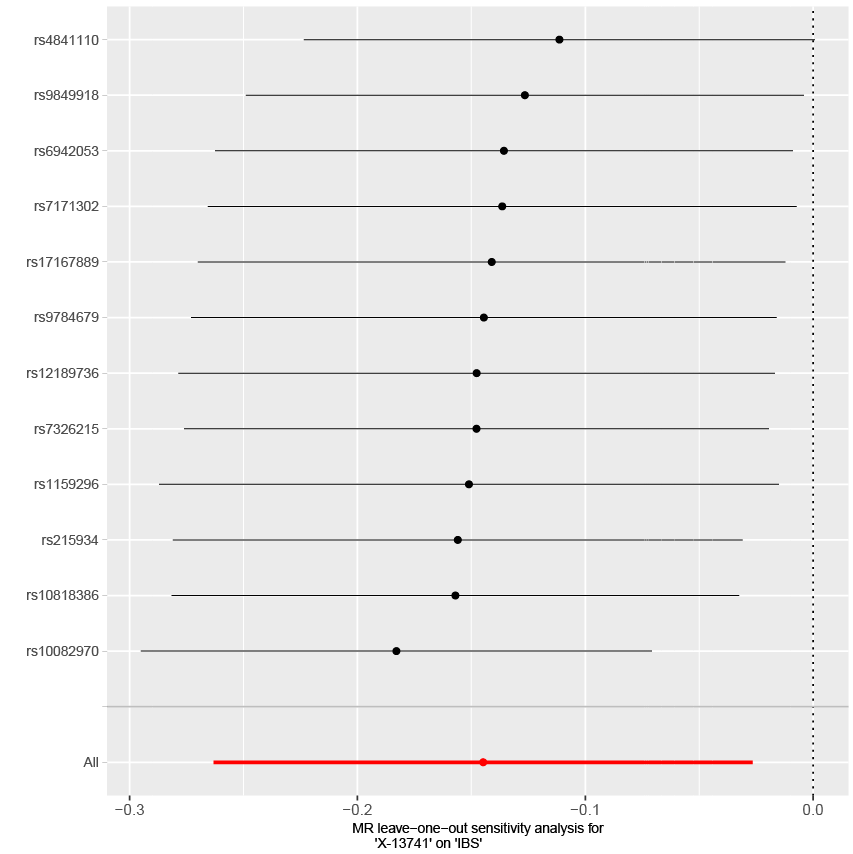


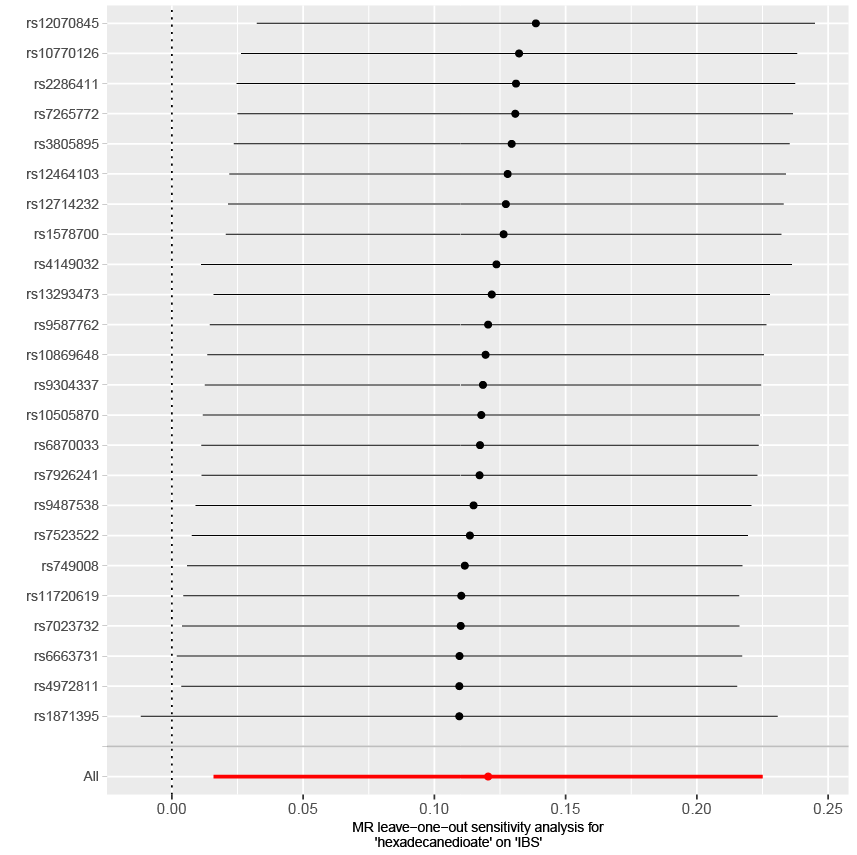


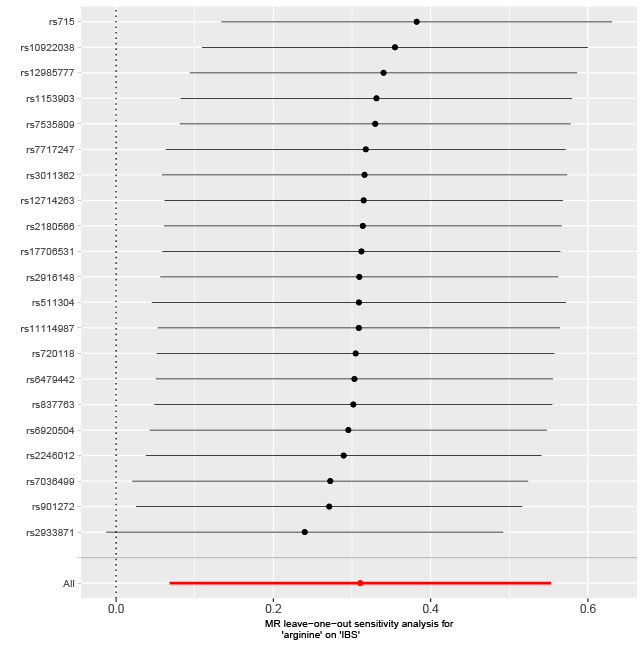

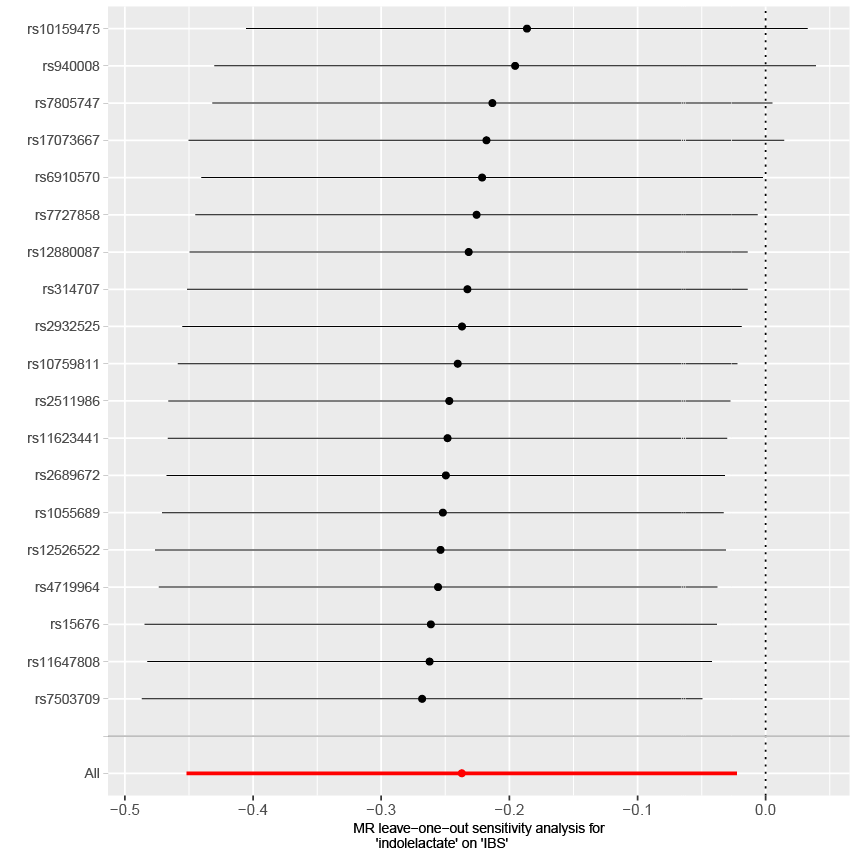


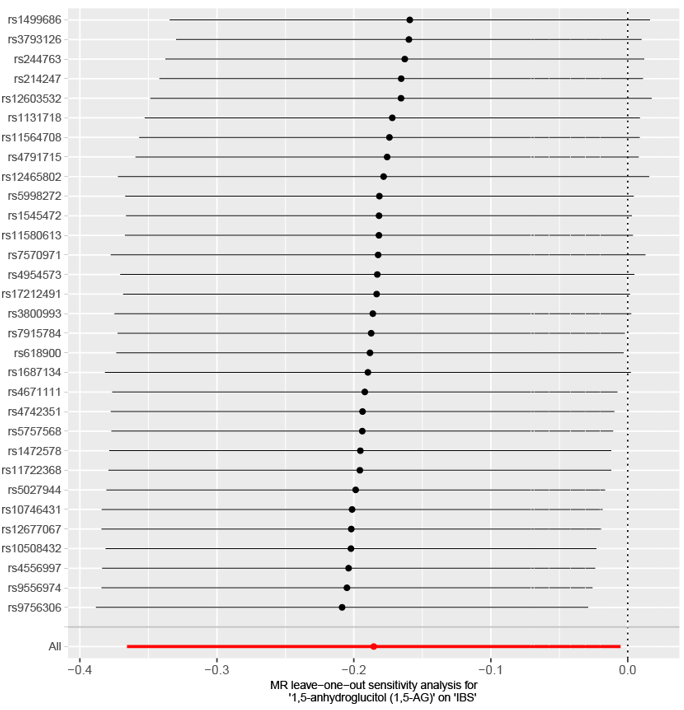


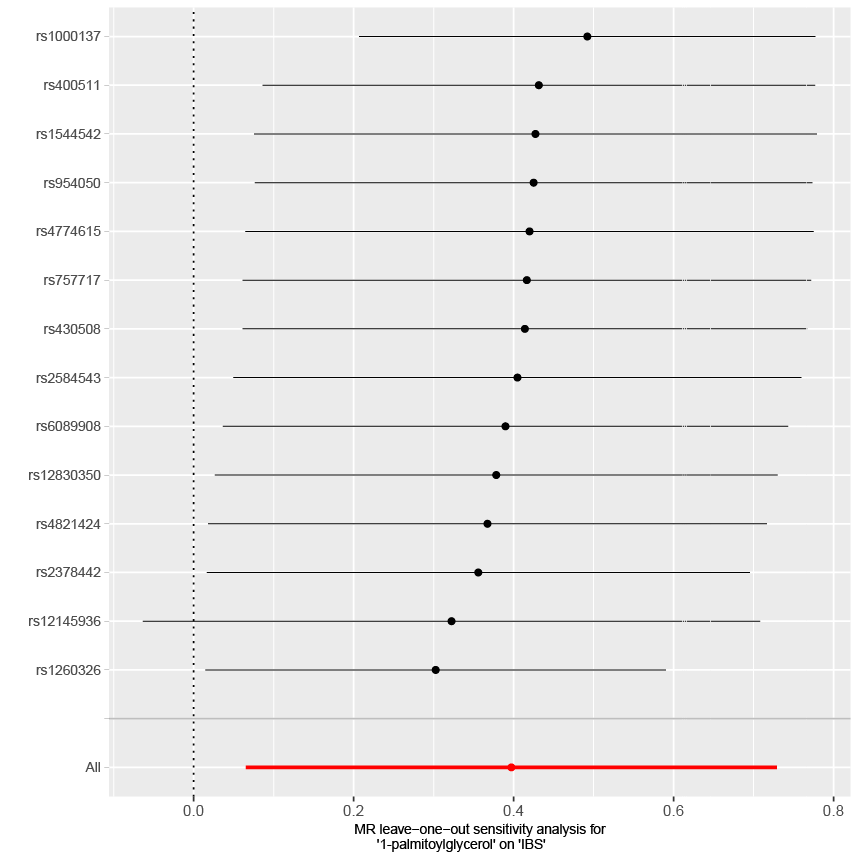


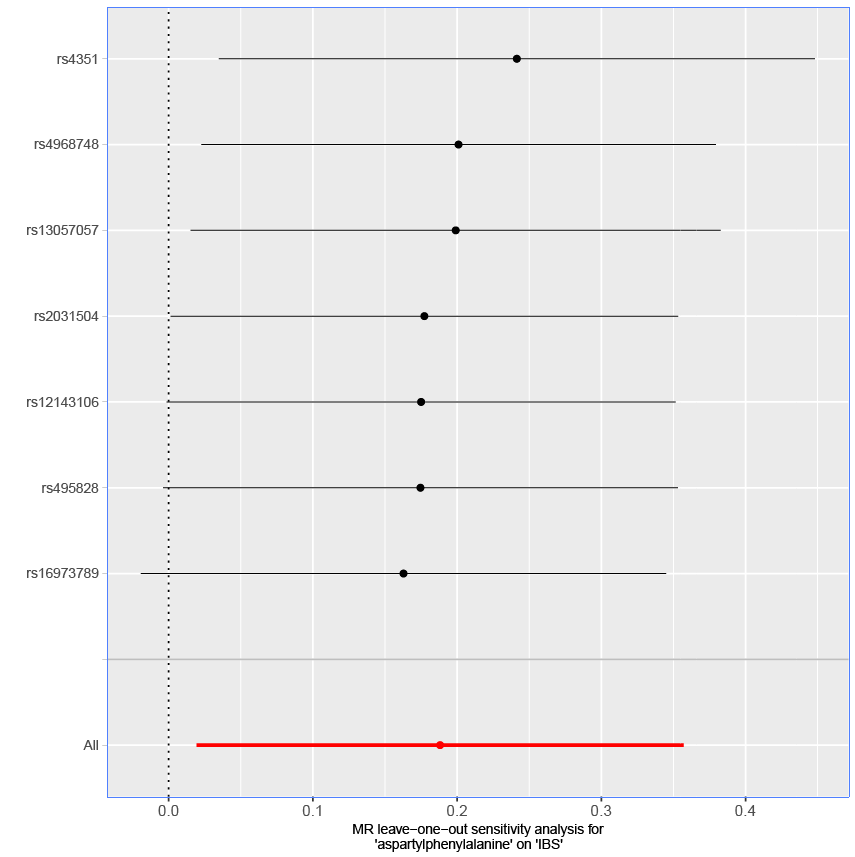


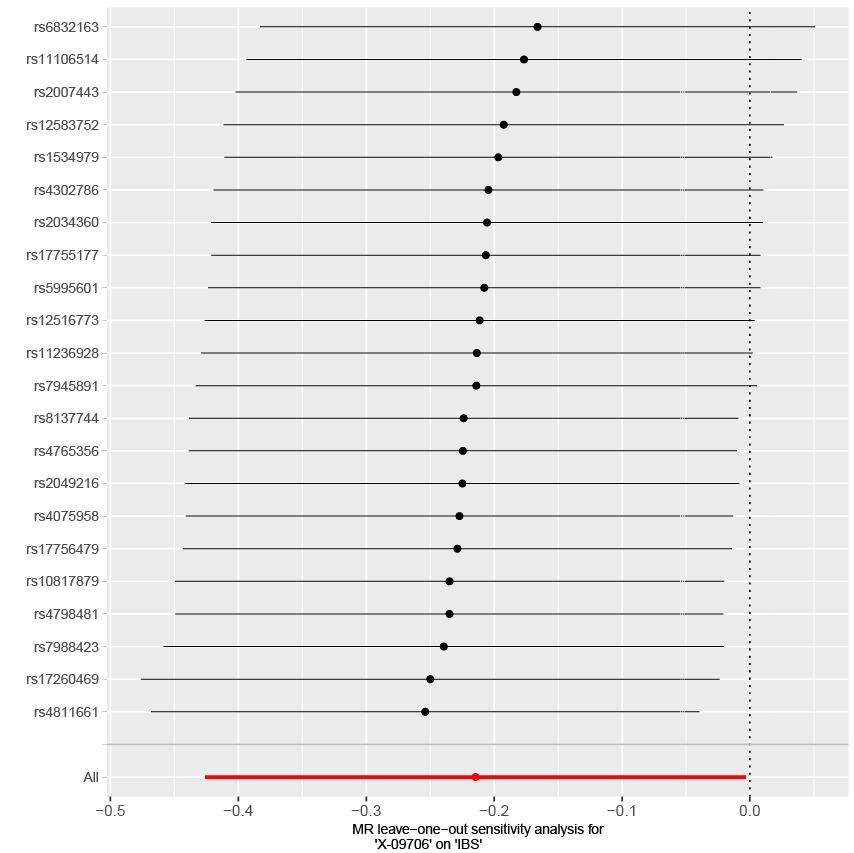


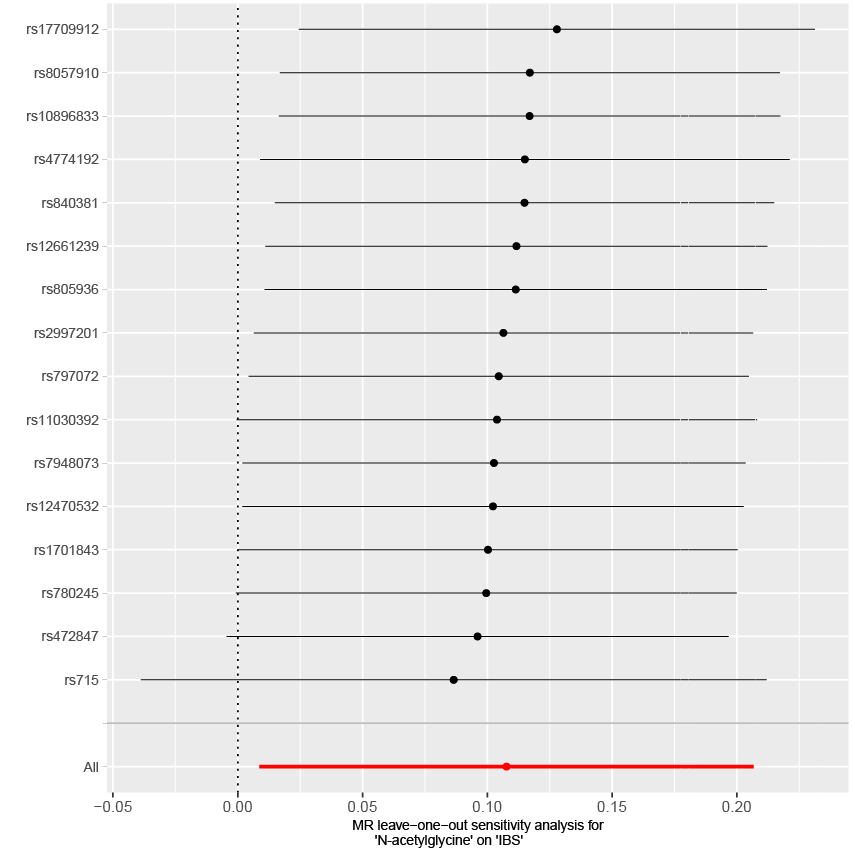


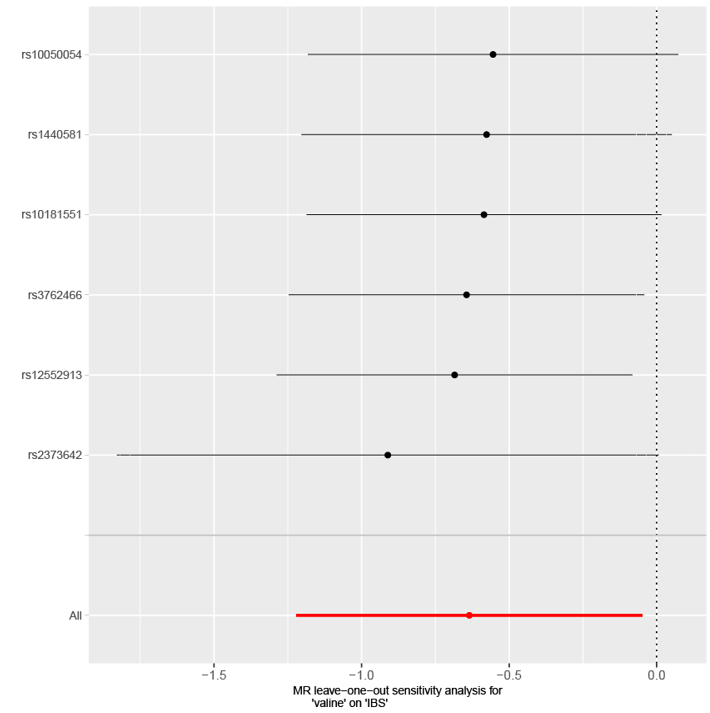


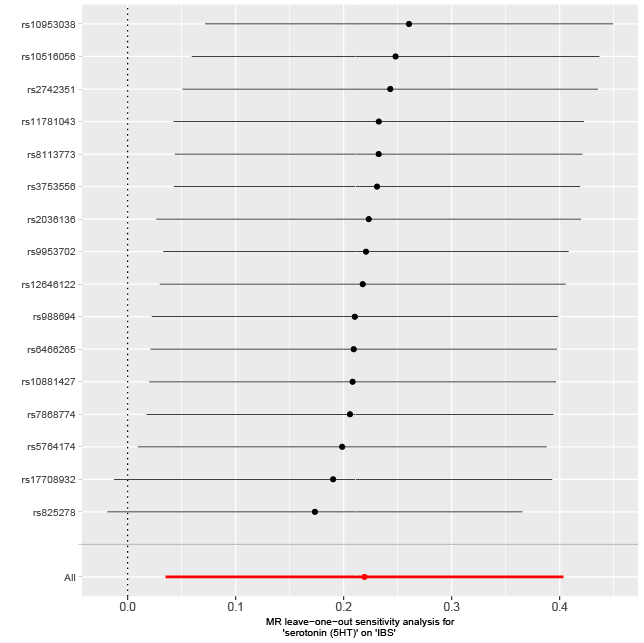


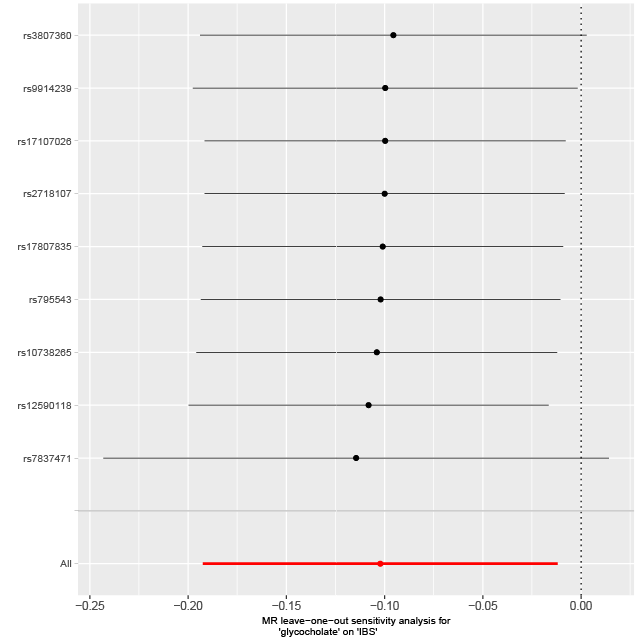


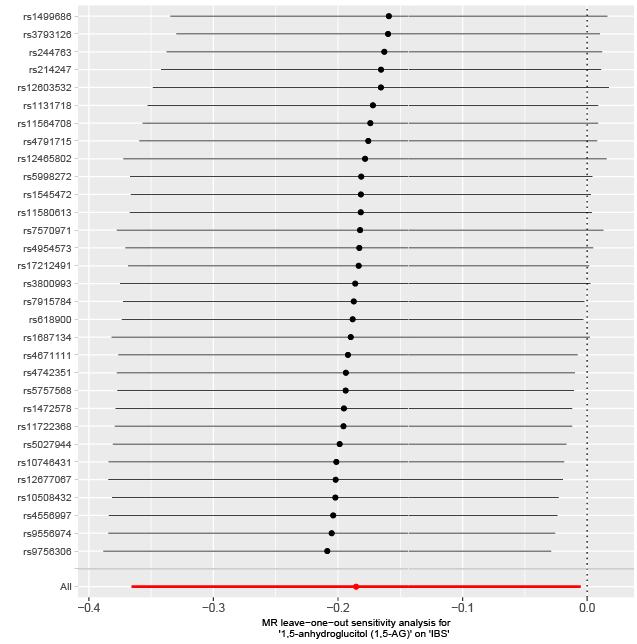


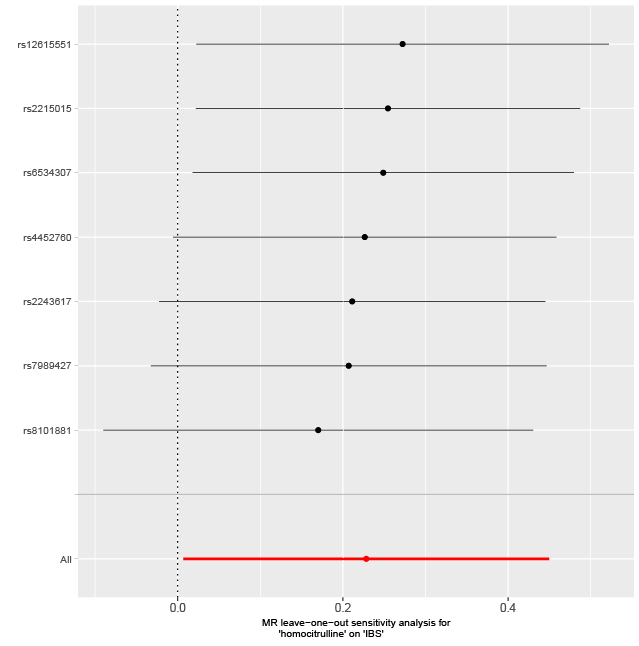


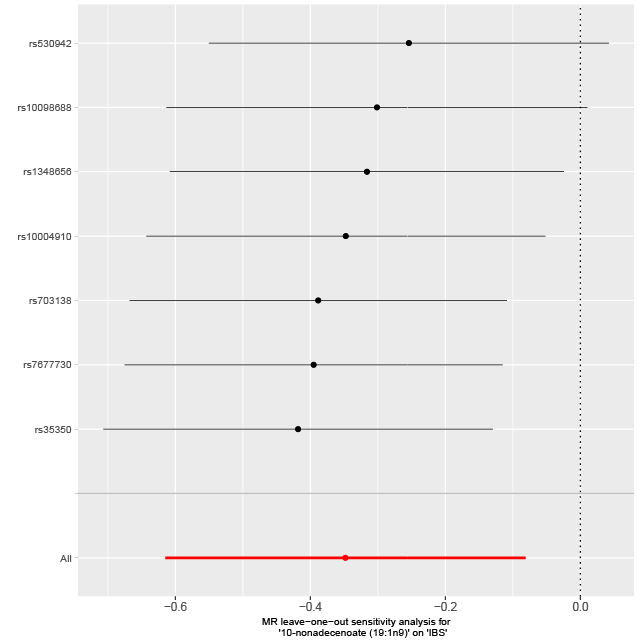


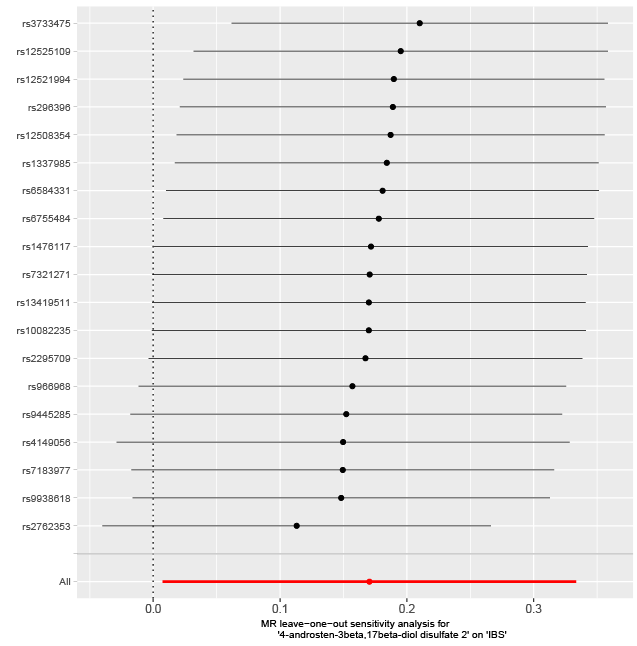


**S2 Fig.** **Meta-analysis of selected metabolites on IBS.**

(a)X-12855


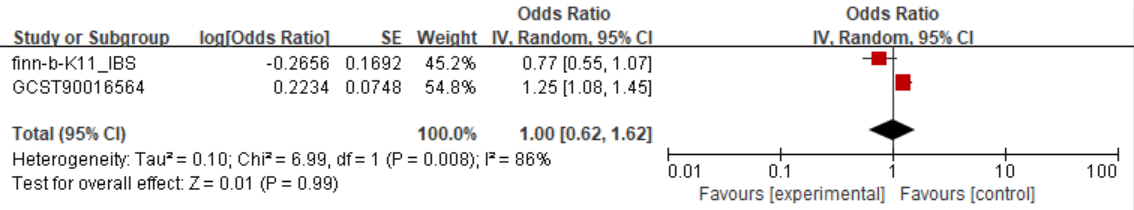


(b) 1-palmitoylglycerophosphocholine


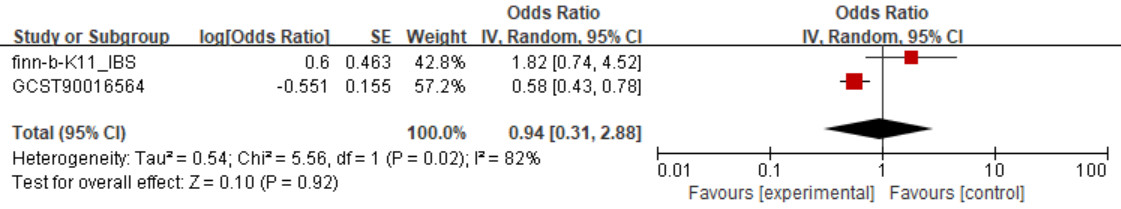


(c) X-14374


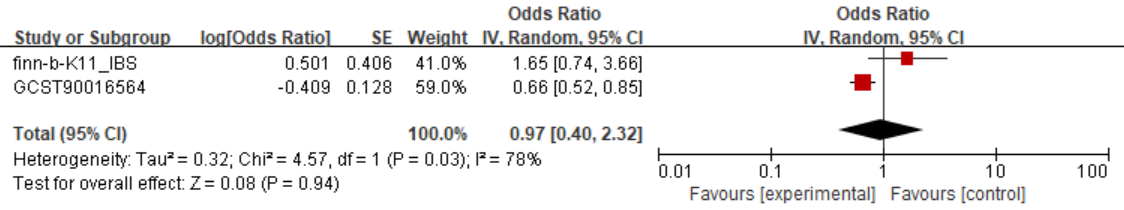


(d) phenol sulfate


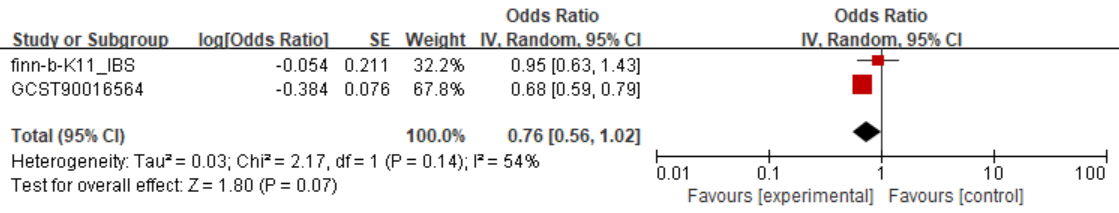

Supplement: S1 File — (DOCX) [file pone.0298963.s008.docx]
